# Mechanical ventilation profile in an adult ICU in Brazil

**DOI:** 10.1186/cc10188

**Published:** 2011-06-22

**Authors:** CSV Barbas, C Saghabi, C Taniguchi, K Timenetsky, S Calegaro, CSA Azevedo, TP Stuchi, RA Caserta, E Silva

**Affiliations:** 1 Hospital Israelita Albert Einstein, São Paulo - SP, Brazil

## Background

Adult critically ill patients need invasive mechanical ventilation support due to distinct causes that vary from an elective high-risk surgery to post cardiorespiratory arrest.

## Objective

To know the mechanical ventilation profile in an adult medical-surgical ICU in Brazil. To study adults patients that needed more than 24 hours invasive mechanical ventilation support in an adult ICU in Brazil.

## Methods

We analyzed all patients that needed more than 24 hours invasive mechanical ventilatory support admitted to Albert Einstein Adult medical-surgical 36-bed ICU from December 2008 to April 2010. We studied patient's age, sex, APACHE II score, cause of intubation/mechanical ventilation, duration of ventilatory support, maximum inspiratory pressure (MIP; mmHg), maximum expiratory pressure (MEP; mmHg) and respiratory shallow breathing index (RSBI; l), rate of extubation success and ratio of reintubation.

## Results

A total of 252 patients were studied, mean age 63 ± 19 years, 35% females, mean APACHE II score 22 ± 6. The main cause of intubation/mechanical ventilation was acute hypoxemic respiratory failure (35%) followed by depressed level of consciousness (34%), post high-risk surgery (19%), airway obstruction (3%), hemodynamic instability (5%) and respiratory fatigue (01%). The mean duration of invasive mechanical ventilation was 114 ± 4 hours (27 to 566 hours). Before a spontaneous breathing trial to check readiness for extubation, mean MIP was 48 ± 12 (20 to 120) mmHg, mean MEP was 45 ± 15 (12 to 120) mmHg and mean RR/TV (l) was 53 ± 20 (5 to 190). The extubation success rate was 87.3%. We used non-invasive ventilation immediately after extubation in 66% of our patients. In total, 12.7% patients needed reintubation. The hospital mortality rate was 8.75% (22 patients). There were no differences in regard to age, gender, mechanical ventilation time, MIP, MEP, RSBI, use of non-invasive mechanical ventilation and reintubation rate between patients that survived and those that died.

## Conclusion

In our ICU the main causes for invasive mechanical ventilatory support were hypoxemic respiratory failure and post high-risk surgery. The mean duration of invasive support was 4.7 days and the reintubation rate was 12.7%.

